# External validation of the 2020 ERC/ESICM prognostication strategy algorithm after cardiac arrest

**DOI:** 10.1186/s13054-022-03954-w

**Published:** 2022-04-11

**Authors:** Chun Song Youn, Kyu Nam Park, Soo Hyun Kim, Byung Kook Lee, Tobias Cronberg, Sang Hoon Oh, Kyung Woon Jeung, In Soo Cho, Seung Pill Choi, the Korean Hypothermia Network Investigators

**Affiliations:** 1 Department of Emergency Medicine, Seoul St. Mary’s Hospital, College of Medicine, The Catholic University of Korea, 222 Banpo-daero, Seocho-gu, Seoul, 137-701 Republic of Korea; 2 Department of Emergency Medicine, Eunpyeong St. Mary Hospital, College of Medicine, The Catholic University of Korea, Seoul, 137-701 South Korea; 3 Department of Emergency Medicine, Chonnam National University Medical School, Gwangju, Korea; 4 Department of Clinical Sciences, Neurology, Skåne University Hospital, Lund University, Lund, Sweden; 5 Department of Emergency Medicine, Hanil General Hospital, Korea Electric Power Medical Corporation, Seoul, Korea; 6 Department of Emergency Medicine, Seoul St. Mary’s Hospital, College of Medicine, The Catholic University of Korea, 222, Banpo-daero, Seocho-Gu, Seoul, 06591 Republic of Korea

**Keywords:** Cardiac arrest, Outcome, Guideline algorithm, Prognostic accuracy

## Abstract

**Purpose:**

To assess the performance of the post-cardiac arrest (CA) prognostication strategy algorithm recommended by the European Resuscitation Council (ERC) and the European Society of Intensive Care Medicine (ESICM) in 2020.

**Methods:**

This was a retrospective analysis of the Korean Hypothermia Network Prospective Registry 1.0. Unconscious patients without confounders at day 4 (72–96 h) after return of spontaneous circulation (ROSC) were included. The association between the prognostic factors included in the prognostication strategy algorithm, except status myoclonus and the neurological outcome, was investigated, and finally, the prognostic performance of the prognostication strategy algorithm was evaluated. Poor outcome was defined as cerebral performance categories 3–5 at 6 months after ROSC.

**Results:**

A total of 660 patients were included in the final analysis. Of those, 108 (16.4%) patients had a good neurological outcome at 6 months after CA. The 2020 ERC/ESICM prognostication strategy algorithm identified patients with poor neurological outcome with 60.2% sensitivity (95% CI 55.9–64.4) and 100% specificity (95% CI 93.9–100) among patients who were unconscious or had a GCS_M score ≤ 3 and with 58.2% sensitivity (95% CI 53.9–62.3) and 100% specificity (95% CI 96.6–100) among unconscious patients. When two prognostic factors were combined, any combination of prognostic factors had a false positive rate (FPR) of 0 (95% CI 0–5.6 for combination of no PR/CR and poor CT, 0–30.8 for combination of No SSEP N20 and NSE 60).

**Conclusion:**

The 2020 ERC/ESICM prognostication strategy algorithm predicted poor outcome without an FPR and with sensitivities of 58.2–60.2%. Any combinations of two predictors recommended by ERC/ESICM showed 0% of FPR.

**Supplementary Information:**

The online version contains supplementary material available at 10.1186/s13054-022-03954-w.

## Introduction

Due to hypoxic-ischemic brain injury, most patients are comatose following resuscitation from out-of-hospital cardiac arrest (OHCA) and are admitted to an intensive care unit (ICU) [1, 2]. Targeted temperature management (TTM) of 32–36 °C is the currently recommended treatment for brain injury after cardiac arrest (CA), but it may affect sedative drug metabolism and make accurate outcome prediction difficult [3–6]. Withdrawal of life-sustaining treatment (WLST) based on a predicted poor neurological outcome is the most common cause of death in patients undergoing TTM after CA [2, 7, 8]. Therefore, strategies for predicting outcome accurately and preventing inaccurate prognostication are critically needed.

The European Resuscitation Council (ERC) and the European Society of Intensive Care Medicine (ESICM) published a 4-step prognostication strategy algorithm in 2015 based on current scientific evidence and expert opinions to minimize the risk of erroneous prognostication [9]. According to two single-center studies and one multicenter study using TTM trial data, the 2015 ERC/ESICM algorithm showed 100% specificity and 18–42% sensitivity [10–12]. However, all of these studies were potentially biased by self-fulfilling prophecy because WLST was permitted for patients with a presumed poor prognosis and was the most common cause of death [13]. A new, more simplified prognostication strategy algorithm was published in 2020 [14]. The Korean Hypothermia Network Prospective Registry 1.0 (KORHN-PRO 1.0) is a web-based registry of OHCA patients treated with TTM between October 2015 and December 2018 [15]. KORHN-PRO 1.0 is an ideal source for validation of the proposed prognostication algorithm due to the minimal practice of WLST in Korean intensive care.

We hypothesized that the 2020 ERC/ESICM prognostication strategy algorithm would predict poor neurological outcome without false positive predictions in OHCA patients treated with TTM in a Korean setting. We performed a retrospective analysis of data to assess the performance of the post-cardiac arrest prognostication strategy algorithm recommended by the ERC/ESICM.

## Methods

### The Korean hypothermia network prospective registry 1.0

KORHN-PRO 1.0 is a multicenter, internet-based registry of OHCA patients treated with TTM irrespective of their initial rhythm between October 2015 and December 2018. The KORHN-PRO registry was registered under clinicaltrials.gov as protocol NCT02827422. Design, participation, data collection and results have previously been published [15]. Briefly, nontraumatic OHCA patients treated with TTM at each center were reported in KORHN-PRO 1.0. The patients with the following conditions were excluded from the registry: active intracranial bleeding, acute stroke, known limitations in therapy including do-not-attempt resuscitation orders, a known prearrest cerebral performance category (CPC) of 3 or 4 and body temperature < 30 °C on admission. Written informed consent was obtained from all patients’ legal surrogates at the participating hospital.

### Definition of poor neurological outcome predictors

The screening criterion of the 2020 ERC/ESICM prognostication strategy algorithm was unconscious patients with a Glasgow Coma Scale Motor (GCS_M) score equal to or below 3 ≥ 72 h after return of spontaneous circulation (ROSC) without confounders. In this study, variables collected on day 4 (72–96 h after ROSC) in the KORHN-PRO 1.0 were considered representative of variables “ ≥ 72 h after ROSC" suggested in the guideline, being closest to the guideline recommendation. Accordingly, a GCS_M score ≤ 3 on day 4 was used as a screening criterion. We defined “awakening” as follows. (1) Eyes open spontaneously or in response to voice requests and followed commands or visually tracked moving objects. (2) GCS_M score is equal to 6 points [16]. Patients who did not meet these criteria were defined as unconsciousness. “Without confounders” is difficult to define due to the nature of a registry-based study. Patients receiving sedatives or NMBs on Day 4 were excluded as “sedated or paralyzed.”

The absence of both pupillary light reflex (PLR) and corneal reflex (CR) on day 4 was defined as no PR/CR [17]. Median nerve somatosensory evoked potentials (SSEPs) performed 24 h after ROSC were used for analysis [18]. A bilateral absence of N20 waves was used as a poor outcome predictor and defined as no SSEP N20.

Electroencephalography (EEG) performed 24 h after ROSC was used for analysis. A neurologist blinded to the patient's outcome retrospectively analyzed raw EEG data based on the terminology of the American Clinical Neurophysiology Society and classified them into highly malignant patterns, malignant patterns and benign patterns [19]. Suppressed background, suppressed background with continuous periodic discharges and burst-suppression background were defined as highly malignant EEG and used as poor outcome predictors [20].

Serum neuron-specific enolase (NSE) levels assessed at each participating hospital at 24, 48 and 72 h after CA were entered in the registry [21]. High NSE levels (NSE 60) were defined as NSE > 60 µg/L at 48 h and/or 72 h after ROSC according to the guidelines. Status myoclonus was not included in the KORHN-PRO 1.0. Most sites included in the KORHN start TTM in the emergency room, and almost all use neuromuscular blocking agents (NMBs) to control shivering. Therefore, it is very difficult to detect status myoclonus as defined in the guidelines.

The indication of neuroimaging was made at discretion of the responsible physicians with various time points. The date was entered into the registry if brain CT and MRI had been examined. However, the results of brain CT and DWI were not entered into the registry. Therefore, all brain CTs and DWIs were collected and additional qualitative analysis was performed. Briefly, neuroradiologist blinded to all clinical information reviewed brain CT and MRI (DW imaging and ADC maps) for each patient using a picture archiving and communication system. The first available brain CT within 72 h after ROSC and brain diffusion-weighted imaging (DWI) between 2 and 7 days after ROSC were analyzed in this study. A neuroradiologist visually assessed whether there was generalized edema in brain CT and brain DWI. In the case of generalized edema, it was judged that there was diffuse and extensive anoxic injury suggested in the 2020 ERC/ESICM guideline, and this was defined as poor CT and poor DWI.

### Outcome

The primary outcome was a poor neurological outcome at 6 months after ROSC, defined as a cerebral performance categories (CPCs) 3–5. The CPC scale ranges from 1 to 5: 1 represents good cerebral performance or slight cerebral disability, 2 represents moderate disability, independence in activities of daily life, 3 represents severe disability, dependence on others for daily support, 4 represents a coma or vegetative state, and 5 represents death or brain death. Follow-up was performed either face-to-face or by telephone.

### Statistical analysis

Categorical variables are presented as counts and percentages, and continuous variables are presented as medians (interquartile ranges, IQRs). To compare differences in patient characteristics and outcomes, a t-test, Fisher’s exact test and the Chi-square test were used.

Two different cohorts were analyzed. The first cohort consisted of unconscious patients ≥ 72 h after ROSC without confounders (*N* = 660) to test the GCS_M score as one variable in the algorithm. The second cohort consisted of the remaining unconscious patients with a GCS_M score ≤ 3 at ≥ 72 h after ROSC without confounders (*N* = 589) to test variables after screening.

The term "true" was defined when the predicted outcome and the reported outcome were the same, and "false" was defined when the predicted outcome was different from the reported outcome. A poor neurological outcome was defined as “positive,” and a good outcome was defined as “negative” [12]. Statistical analyses were performed using SPSS 20.0 (Chicago, IL) and MedCalc15.2.2 (MedCalc Software, Mariakerke, Belgium). *P* values ≤ 0.05 were considered indicative of statistical significance.

## Results

### Patients

Between October 2015 and December 2018, 1373 OHCA subjects were registered in the KORHN-PRO 1.0. Of those, 328 were excluded due to missing data on the primary outcome (*n* = 34), WLST (*n* = 12), missing data on the GCS_M at day 4 (*n* = 14), death before day 4 (*n* = 256) and discharge before day 4 (*n* = 12). The causes of death for patients who died before day 4 were as follows: cardiovascular (*n* = 92 (35.9%)), cerebral (*n* = 28 (10.9%)), multiorgan failure (*n* = 120 (46.9%)) and other or undetermined (*n* = 15 (5.9%)). Demographic characteristics of subjects who died before Day 4 and those who did not are presented in Additional file 1: Table S1. The length of stay and outcome predictors of 12 patients with WLST are shown in Additional file 1: Table S2.

**Fig. 1:**
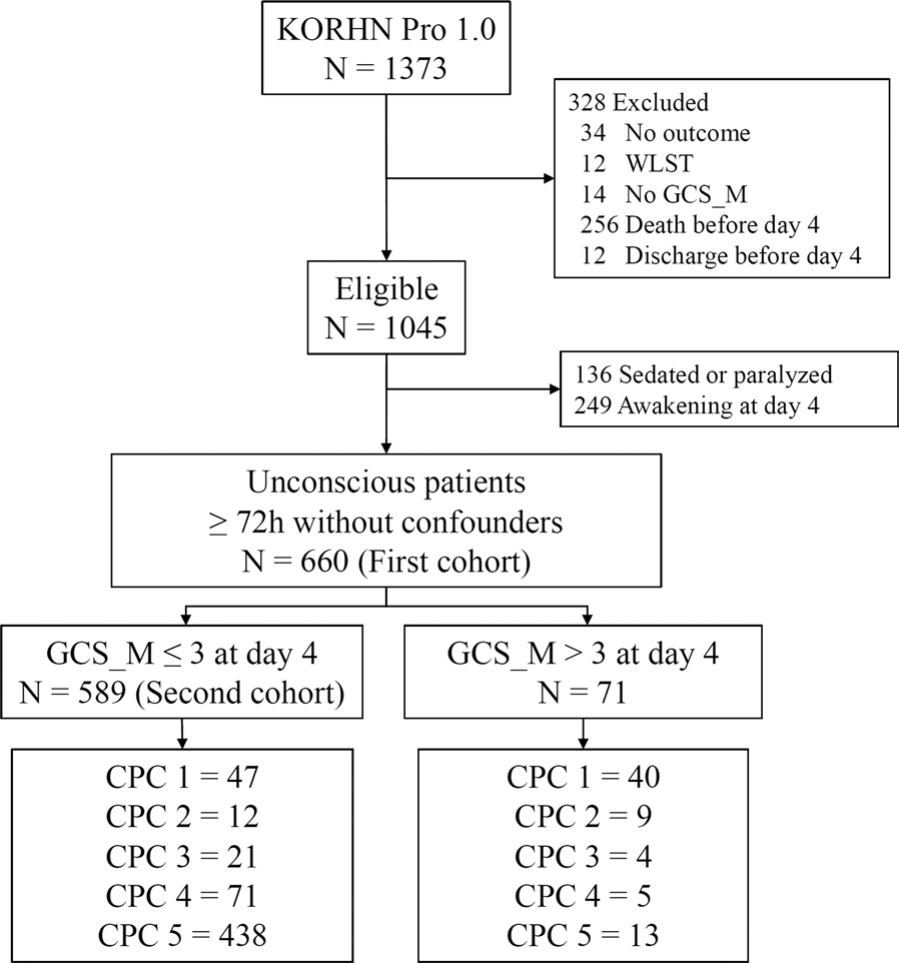
Flow diagram of included patients. Unconscious patients ≥ 72 h after ROSC without confounders (*N* = 660) were defined as the first cohort to test the GCS_M score as one variable in the algorithm. Unconscious patients with a GCS_M score ≤ 3 at ≥ 72 h after ROSC without confounders (*N* = 589) were defined as the second cohort

Three hundred eighty-five subjects were further excluded because they were sedated, paralyzed (*n* = 136) or awakened on day 4 (*n* = 249). Thus, 660 subjects met the inclusion, but not the exclusion, criteria, and 108 (16.4%) of them had good outcomes (first cohort). Of the included subjects, 589 had a GCS_M score ≤ 3 on day 4 (second cohort) [Fig. 1].

The demographic characteristics of the subjects according to the primary outcome are shown in Table 1. The prognostic investigations suggested in the guidelines were performed with the following results: 518 (78.5%) patients with both PLR and CR, 150 (22.7%) with median nerve SSEP, 363 (55.0%) with NSE, 249 (37.7%) with EEG, 602 (91.2%) with brain CT and 332 (50.3%) with brain DWI. There were no significant differences regarding time to SSEP, time to EEG, time to brain CT or time to brain DWI between the two outcome groups.

**Table 1:** Demographic characteristics of subjects according to neurological outcome

	Good *N* = 108	Poor *N* = 552	*p*
Age, median (IQR)	57 (47–66)	61 (50–72)	0.003
Sex, male	88 (81.5)	362 (65.6)	0.001
Past history			
HTN	37 (34.3)	319 (39.7)	0.291
DM	22 (20.4)	157 (28.4)	0.084
CA place, residence, yes	41 (38.0)	313 (56.7)	< 0.001
Witnessed arrest, no. (%)	84 (77.8)	312 (56.5)	< 0.001
Bystander CPR, no. (%)	72 (66.7)	327 (59.2)	0.149
Shockable rhythm, no. (%)	71 (65.7)	90 (16.3)	< 0.001
Cardiac cause of arrest, no. (%)	93 (86.1)	253 (45.8)	< 0.001
Minutes to ROSC, median (IQR)	16.0 (10.5–19.0)	29.0 (19.5–40.0)	< 0.001
Outcome predictors			
Neurological examination, *n*	71	447	< 0.001
No pupillary and corneal reflex, yes	0 (0)	281 (62.9)	
NSE, 48 and/or 72 h, *n*	56	307	< 0.001
NSE > 60, yes	3 (5.4)	242 (78.8)	
SSEP, ≥ 24 h, *n*	18	132	
Bilaterally absent N20, yes	0 (0)	96 (72.7)	< 0.001
Time to SSEP, h	76.0 (67.5–98.0)	72.0 (60.5–95.5)	0.731
EEG, ≥ 24 h, *n*	43	206	
Highly malignant EEG, yes	0 (0)	122 (59.2)	< 0.001
Time to EEG, h	37.9 (25.2–65.4)	66.4 (41.8–89.1)	0.100
Brain CT, ≤ 72 h, *n*	99	503	
Poor CT, yes	9 (9.1)	165 (32.8)	< 0.001
Time to Brain CT, min	38.0 (28.0–57.5)	61.5 (22.5–113.5)	0.301
Brain DWI, 2–7 day, *n*	50	282	
Poor DWI, yes	0 (0)	220 (78.0)	< 0.001
Time to Brain DWI, h	83.0 (76.0–104.5)	78.0 (68.0–85.0)	0.145

Variables are expressed as median (interquartile range) or n (%)

IQR, interquartile range; HTN, hypertension; DM, diabetes mellitus; CA, cardiac arrest; CPR, cardiopulmonary resuscitation; ROSC, return of spontaneous circulation; NSE, neuron-specific enolase; SSEP, somatosensory evoked potential; EEG, electroencephalogram; CT, computed tomography; DWI, diffusion-weighted image

Of 660 patients included in this study, 87 (13.2%) had CPC1, 21 (3.2%) had CPC2, 25 (3.8%) had CPC3, 76 (11.5%) had CPC4, and 451 (68.3%) had CPC5 at 6 months.

### Prognostic values of single predictors

The areas under the receiver operating characteristic (ROC) curves, sensitivities and specificities of single predictors in the first cohort are shown in Table 2. No PR/CR, SSEP N20, highly malignant EEG and poor DWI all showed 100% specificity for poor neurological outcome, and among them, the sensitivity of poor DWI was the highest at 78.0% (95% CI 72.7–82.7). On the other hand, the specificities of NSE 60 and poor CT were 94.6% (95% CI 85.1–98.9) and 90.9% (95% CI 83.4–95.8), respectively, and the cutoff value to maintain 100% specificity was 88 for NSE.

**Table 2:** Prognostic performance of single prognostic methods as recommended by ERC/ESICM

	*N*	AUC	Sensitivity	Specificity	TP	TN	FP	FN
A. Unconscious patients at ≥ 72 h after ROSC without confounders								
GCS_M ≤ 3	660	0.707 (0.644–0.770)	89.9 (87.3–92.3)	69.0 (56.9–79.5)	530	49	22	59
No PR/CR	518	0.661 (0.588–0.734)	62.9 (58.2–67.4)	100 (94.9–100)	281	71	0	166
No SSEP N20	150	0.864 (0.805–0.923)	72.7 (64.3–80.1)	100 (81.5–100)	96	18	0	36
NSE > 60	363	0.867 (0.822–0.912)	78.8 (73.8–83.3)	94.6 (85.1–98.9)	242	53	3	65
Highly malignant EEG	249	0.796 (0.741–0.851)	59.2 (52.2–66.0)	100 (91.8–100)	122	43	0	84
Poor CT	602	0.619 (0.564–0.673)	32.8 (28.7–37.1)	90.9 (83.4–95.8)	165	90	9	338
Poor DWI	332	0.890 (0.856–0.924)	78.0 (72.7–82.7)	100.0 (92.9–100)	220	50	0	62
B. Unconscious patients with GCS_M ≤ 3 at ≥ 72 h after ROSC without confounders								
No PR/CR	471	0.821 (0.778–0.864)	64.1 (59.4–68.7)	100 (91.0–100)	277	39	0	155
No SSEP N20	133	0.874 (0.799–0.949)	74.8 (66.3–82.1)	100 (54.1–100)	95	3	0	32
NSE 60	319	0.905 (0.870–0.939)	81.0 (76.0–85.3)	100 (86.3–100)	238	25	0	56
Highly malignant EEG	224	0.802 (0.738–0.866)	60.4 (53.3–67.2)	100 (84.6–100)	122	22	0	80
Poor CT	537	0.601 (0.527–0.674)	33.6 (29.4–38.0)	86.5 (74.2–94.4)	163	45	7	322
Poor DWI	298	0.897 (0.860–0.934)	79.5 (74.2–84.1)	100 (86.3–100)	217	25	0	56

AUC, area under the curve; TP, true positive; TN, true negative; FP, false positive; FN, false negative; PR, pupillary light reflex; CR, corneal reflex; SSEP, somatosensory evoked potential; NSE, neuron-specific enolase; EEG, electroencephalogram; CT, computed tomography; DWI, diffusion-weighted image

The areas under the ROC curves, sensitivities and specificities of single predictors in the second cohort are shown in Table 2. The specificities of all predictors except poor CT were 100%. The area under the ROC curve and sensitivity of NSE 60 were the highest among the predictors (AUC 0.905, sensitivity 81%, 95% CI 76.0–85.3).

### Prognostic values of combined predictors

Table 3 shows the sensitivities and specificities for single and combinations of two predictors in the first cohort and the second cohort. All combinations of two predictors in the first cohort showed 100% specificity, and the sensitivity was 26.3–76.6%. The sensitivity of the combination of NSE 60 and poor DWI in the first cohort was the highest at 76.6% (95% CI 69.2–82.9).

**Table 3:** Sensitivities and specificities for single and combined predictors in the ERC/ESICM algorithm

	GCS_M ≤ 3	PR/CR	SSEP	EEG	NSE	Poor CT	Poor DWI
A. Unconscious patients at ≥ 72 h after ROSC without confounders							
GCS_M ≤ 3	89.9/69.0 (660)						
No PR/CR	64.1/100 (471)	62.86/100 (518)					
No SSEP N20	74.8/100 (133)	59.09/100 (122)	72.73/100 (150)				
Highly malignant EEG	60.4/100 (224)	45.12/100 (192)	56.63/100 (94)	59.22/100 (249)			
NSE 60	81.0/100 (319)	54.17/100 (298)	76.47/100 (95)	60.63/100 (153)	78.83/94.64 (363)		
Poor CT	33.6/86.5 (537)	26.3/100 (478)	31.6/100 (135)	26.5/100 (224)	33.5/100 (341)	32.8/90.9 (602)	
Poor DWI	83.2/92.0 (298)	58.2/100 (257)	68.5/100 (124)	49.0/100 (178)	76.6/100 (188)	31.7/100 (314)	78.0/100 (332)
B. Unconscious patients with GCS_M ≤ 3 at ≥ 72 h after ROSC without confounders							
No PR/CR		64.1/100 (471)					
No SSEP N20		60.8/100 (111)	74.8/100 (133)				
Highly malignant EEG		46.3/100 (176)	58.0/100 (85)	60.4/100 (224)			
NSE 60		55.5/100 (271)	79.3/100 (85)	62.1/100 (137)	81.0/100 (319)		
Poor CT		27.2/100 (435)	32.7/100 (119)	26.9/100 (201)	34.9/100 (299)	33.6/86.5 (537)	
Poor DWI		59.7/100 (238)	71.2/100 (110)	50.3/100 (161)	79.6/100 (166)	32.8/100 (281)	79.5/100 (298)

The upper column indicates the sensitivity and specificity as a percentage in order. Parentheses indicate the number of patients tested

ROSC, return of spontaneous circulation; PR, pupillary light reflex; CR, corneal reflex; SSEP, somatosensory evoked potential; EEG, electroencephalogram; NSE, neuron-specific enolase; CT, computed tomography; DWI, diffusion-weighted image

All combinations of two predictors in the second cohort showed 100% specificity, and the sensitivity was 27.2–79.6%. The sensitivity of the combination of NSE 60 and poor DWI in the second cohort was the highest at 79.6% (95% CI 72.3–85.7).

### Prognostic performance of the 2020 ERC/ESICM prognostication strategy algorithm

**Fig. 2:**
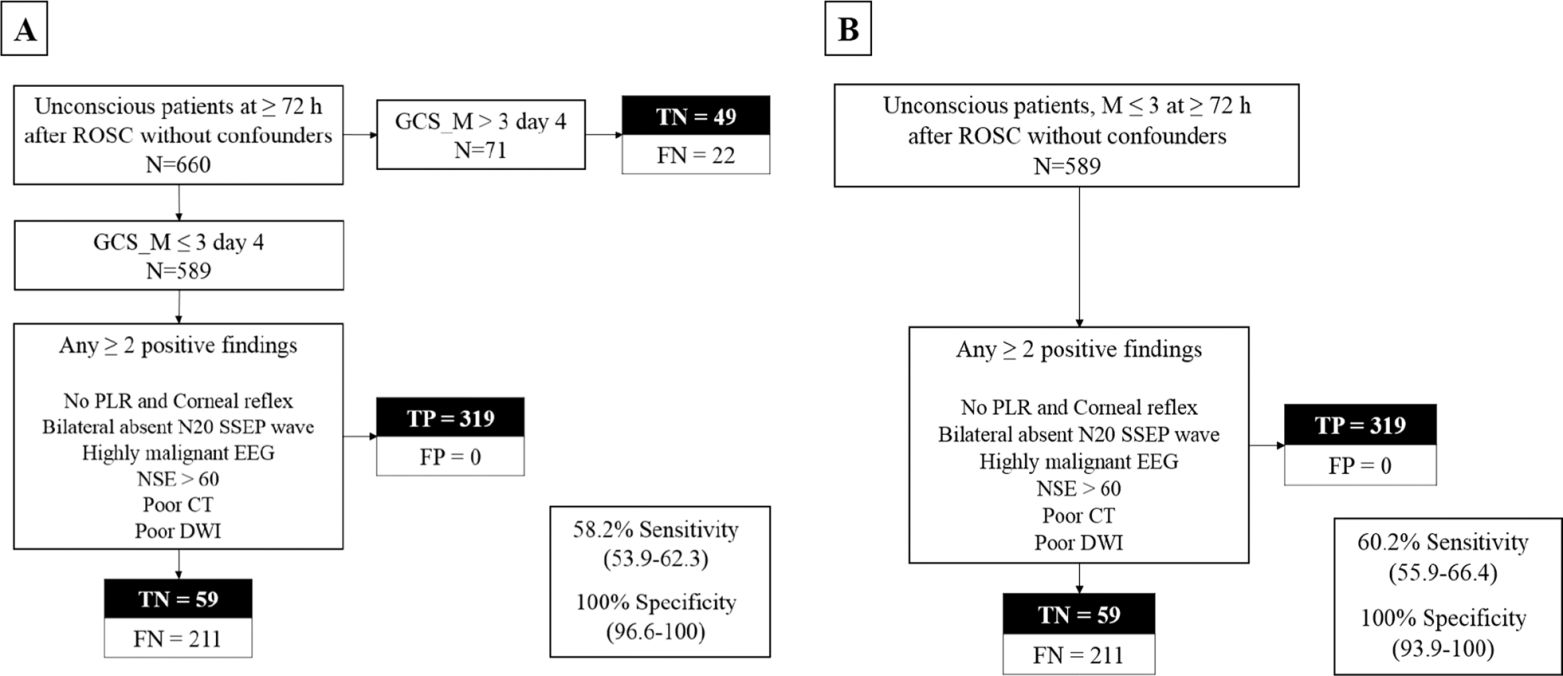
Prognostic performance of the 2020 ERC/ESICM prognostication strategy algorithm in cardiac arrest patients treated with targeted temperature management. **A** The 2020 ERC/ESICM prognostication strategy algorithm for the first cohort. The 2020 ERC/ESICM prognostication strategy algorithm predicted poor outcome without a false positive rate (FPR) and with sensitivities of 58.2%. **B** The 2020 ERC/ESICM prognostication strategy algorithm for the second cohort. The 2020 ERC/ESICM prognostication strategy algorithm predicted poor outcome without a false positive rate (FPR) and with sensitivities of 60.2%

Figure 2 shows the prognostic performance of the 2020 ERC/ESICM prognostication strategy algorithm in the first and second cohorts. The 2020 ERC/ESICM prognostication strategy algorithm predicted poor outcome without a false positive rate (FPR) and with sensitivities of 58.2% in first cohort and 60.2% in second cohort.

## Discussion

The main finding of this study is that the 2020 ERC/ESICM prognostication strategy algorithm predicted poor outcome without false positive prediction and had sensitivities of 58.2–60.2% regardless of whether the GCS_M score was included in the algorithm. Additionally, the combination of SSEP N20 and poor DWI showed the highest sensitivity (76.6% (95% CI 69.2–82.9) in the first cohort, 79.6% (95% CI 72.3–85.7) in the second cohort) without false positive predictions in both cohorts.

Our study has several strengths compared with previous studies [10–12]. First, WLST is uncommon in Korea, which can minimize the self-fulfilling prophecy. There were 12 registered incidents of WLST in the KORHN-PRO 1.0, which accounted for 0.9% of all enrolled patients [Additional file 1: Table S2]. KORHN-PRO 1.0 has a high proportion of CPC4 patients. Of 1373 patients included in the KORHN-PRO 1.0, 132 (9.6%) had CPC3-4 at 6 months and 101 (15.3%) of 660 patients included in this study had CPC3-4 at 6 months. This is very different from studies such as TTM and TTM2 trial, which is clear evidence that WLST is not well implemented in Korea. So, WLST was not a major confounder for self-fulfilling prophecy in this study. Second, this study was conducted on an Asian population with CA with mixed etiologies not limited to cardiac causes of CA. Third, more neurological examinations and imaging tests for neurological outcome prediction were performed than in previous studies. This may be the reason why our study showed high sensitivity compared to previous studies.

Pouplet et al. reported that when neurofilament light chain was added to the ERC/ESICM algorithm using ISOCRATE trial data, the sensitivity was increased while maintaining the specificity at 1 [22]. Although the ISOCRATE trial only enrolled shockable cardiac arrest and had a relatively small sample size, the 2020 ERC/ESICM prognostication strategy algorithm showed 53% sensitivity and 100% specificity, which is consistent with the results of our study.

According to the 2020 ERC/ESICM prognostication strategy algorithm, prognostic assessment should start with an accurate clinical examination after excluding confounding factors such as residual sedation and the effect of muscle relaxants (Step 0) [14]. In Step 1, the patient’s best motor response to painful stimuli (GCS_M) is evaluated as an entry point. In Step 2, “poor outcome likely” will be considered if there are 2 or more pathological findings of the following 6 predictors: no pupillary and corneal reflexes at ≥ 72 h, bilaterally absent N20 SSEP wave, highly malignant EEG > 24 h, NSE > 60 μg/L at 48 h and/or 72 h, status myoclonus ≤ 72 h, diffuse and extensive anoxic injury on brain CT/MRI. Therefore, step 0 corresponds to the first cohort of this study, and step 2 corresponds to the second cohort.

The unconsciousness without confounders in PCAS patients is hard to define because most of the patients require the use of sedatives or NMBs for TTM. In addition, drug clearance varies depending on the patient’s clinical condition. Unconsciousness may be exhibited due to metabolic derangement without brain damage. The World Brain Death Project (WBDP) consensus group recommends that clinical examination be delayed until 5 elimination half-lives of the drug elimination with the longest half-life which is also recommended in 2020 ERC/ESICM guideline especially this is being used to make a WLST decision. Propofol has a half-life of 2.3–4.7 h, which implies the need to stop sedatives for at least 24 h in most cases. It is hard to define “Without confounders” due to the nature of a registry-based study. Therefore, patients who received sedatives or NMBs on Day 4 were excluded as “sedated or paralyzed.” A prospective study is needed to overcome these issues.

The Glasgow Coma Scale Motor Score is a screening criterion for the ERC/ESICM prognostication strategy algorithm due to its relatively high sensitivity for predicting poor neurological outcome. A GCS_M score ≤ 2 was the entry point of the 2015 prognostication algorithm [9]. However, it has been changed to a GCS_M score ≤ 3 (absent, abnormal extension or abnormal flexion) in the 2020 ERC/ESIC guideline because using a GCS_M score ≤ 3 as a screening criterion increases the sensitivity for the prediction of poor neurological outcome without reducing specificity [12, 23]. Our results confirm that a GCS_M score ≤ 3 has high sensitivity (89.9%, 95% CI 87.3–92.3) but low specificity (69.0%, 95% CI 56.9–79.5) for predicting poor neurological outcome. Therefore, the GCS_M score should not be used as a poor outcome predictor but only as a screening criterion.

Predicting neurological outcome with a single predictor can induce false positives, which was confirmed in our study. Our results provide further evidence that a multimodal approach could reduce erroneous prognostication [24, 25]. In particular, caution is required to predict the patient's outcome using NSE and brain CT because 3 patients with NSE 60 and 9 patients with poor CT were false positives in the first cohort.

NSE and EEG have been widely studied to predict neurological outcomes after CA [26, 27]. However, the cutoff values of NSE suggested for each study varied from 33 to 120 µg/L. In addition, the definition of the universally accepted poor outcome predictor using the EEG pattern was different for each study. The 2020 ERC/ESICM guideline presented new definitions of poor outcome predictors for NSE and EEG. In our study, NSE 60 had a specificity of 94.6% in the first cohort and a specificity of 100% in the second cohort. Highly malignant EEG showed 100% specificity in both the first and second cohorts. Moreover, both combinations of predictors using NSE 60 or highly malignant EEG showed 100% specificity. Therefore, our study supports the definition of poor outcome predictors for NSE and EEG presented in the 2020 ERC/ESICM guidelines.

Brain CT and DWI have also been studied extensively to date, but there is no universally accepted standard analysis technique [28]. Neuroimaging can be analyzed either qualitatively or quantitatively. Qualitative analysis has the disadvantage of providing subjective information. One qualitative study using multicenter registry data from brain DWI showed an FPR of 14% [29]. However, there is no cutoff value that is accepted worldwide in quantitative analysis of CT and DWI [30–32]. Therefore, we selected a qualitative analysis method for brain imaging. According to the substudy of the TTM trial, generalized edema according to the local radiologist’s reading at each site could predict poor outcome with sensitivity of 33.6% and specificity of 98.4% [33]. Moreover, CT after 24 h showed higher specificity than CT before 24 h. However, most of the CT scans in our study were performed within 24 h. So, poor CT showed a high FPR in both the first and second cohorts in this study. In contrast, poor DWI showed an FPR of 0% in both cohorts. Therefore, poor CT is not appropriate for diffuse and extensive anoxic injury on brain CT, as suggested in the guidelines, and a new definition is needed. This is an issue that needs to be addressed in future research.

Our study has several limitations. First, this is a retrospective study that should be confirmed by a prospective multicenter study. Second, as mentioned before, status myoclonus was not included from the analysis. Therefore, our study does not fully validate the 2020 ERC/ESICM prognostication strategy algorithm. Third, outcome predictors were not performed according to a prospective protocol but were performed by attending physicians at each institution as needed. Therefore, the possibility of selection bias cannot be excluded. Nevertheless, it is a clear limitation that neurological examination was only performed in 518 patients (78.5%). Fourth, outcome predictors were not blinded to physicians. Therefore, self-fulfilling prophecy cannot be completely ruled out. However, since WLST was minimally implemented, our study minimized the self-fulfilling prophecy. Finally, we used a qualitative analysis for neuroimaging, which may not be common at many institutions. Therefore, it may be difficult to apply our findings.

## Conclusion

The 2020 ERC/ESICM prognostication strategy algorithm predicted poor outcome without an FPR and with sensitivities of 58.2–60.2%. Any combinations of two predictors recommended by ERC/ESICM showed 0% of FPR (95% CI 0–5.6 for combination of no PR/CR and poor CT, 0–30.8 for combination of No SSEP N20 and NSE 60).

## Supplementary Information

**Additional file 1: Supplementary Table 1.** Demographic characteristics of subjects who died before Day 4 and those who did not. **Supplementary Table 2.** Length of stay and outcome predictors of WLST patients.

## Data Availability

The datasets used and/or analyzed during the current study are available from the corresponding author on reasonable request.

## Declarations

### Take-home message

The 2020 ERC/ESICM algorithm reliably predicted poor outcome in patients treated with targeted temperature management after out-of-hospital cardiac arrest where withdrawal of life-sustaining therapy is uncommon. However, our results should be validated by prospective studies.
