# Supplementary material for: External validation of the 2020 ERC/ESICM prognostication strategy algorithm after cardiac arrest
Source: Crit Care. 2022 Apr 11;26:95. doi: 10.1186/s13054-022-03954-w (PMC8996564; doi:10.1186/s13054-022-03954-w)
Supplement: Supplementary file 1 — Additional file 1: Supplementary Table 1. Demographic characteristics of subjects who died before Day 4 and those who did not. Supplementary Table 2. Length of stay and outcome predictors of WLST patients. [file 13054_2022_3954_MOESM1_ESM.docx]

**Supplementary Table 1. Demographic characteristics of subjects who died before Day 4 and those who did not.**

|  | Early Death  N=256 | No early death  N=1117 | p |
| --- | --- | --- | --- |
| Age, median (IQR) | 67 (54-76) | 58 (47-69) | < 0.001 |
| Sex, male | 182 (71.1) | 794 (71.1) | 1.000 |
| Past History  HTN  DM | 109 (42.6)  86 (33.6) | 383 (34.3)  247 (22.1) | 0.014  < 0.001 |
| CA place, residence, yes | 142 (55.5) | 556 (49.8) | 0.111 |
| Witnessed arrest, No. (%) | 157 (61.3) | 747 (66.9) | 0.093 |
| Bystander CPR, No. (%) | 141 (55.1) | 701 (62.8) | 0.027 |
| Shockable rhythm, No. (%) | 45 (17.6) | 415 (37.2) | < 0.001 |
| Cardiac cause of arrest, No. (%) | 154 (60.2) | 697 (62.4) | 0.521 |
| Minutes to ROSC, median (IQR) | 41.0 (32.0-54.8) | 27.0 (16.0-40.0) | < 0.001 |

Variables are expressed as median (interquartile range) or n (%).

Abbreviations: IQR; interquartile range, HTN; hypertension, DM; diabetes mellitus, CA; cardiac arrest, CPR; cardiopulmonary resuscitation, ROSC; return of spontaneous circulation

**Supplementary Table 2. Length of stay and outcome predictors of WLST patients**

|  | LOS | no PR/CR | SSEP N20 | NSE  (µg/L) | EEG | Poor CT | Poor DWI |
| --- | --- | --- | --- | --- | --- | --- | --- |
| 1 | 12 | No | Not checked | 291.2 | Not checked | no | yes |
| 2 | 9 | Yes | Bilaterally absent | 500.0 | malignant | yes | yes |
| 3 | 5 | No | Bilaterally absent | 500.0 | malignant | no | Not checked |
| 4 | 15 | Yes | Bilaterally absent | 500.0 | Highly malignant | yes | yes |
| 5 | 5 | Yes | Bilaterally absent | 493.8 | Highly malignant | yes | yes |
| 6 | 8 | Yes | Bilaterally absent | 213.1 | Highly malignant | no | yes |
| 7 | 8 | Yes | Bilaterally absent | 438.2 | Highly malignant | no | yes |
| 8 | 5 | Yes | Bilaterally absent | 432.2 | Highly malignant | no | yes |
| 9 | 4 | No | Not checked | 84.9 | malignant | no | Not checked |
| 10 | 3 | Yes | Not checked | 332.9 | Not checked | no | yes |
| 11 | 9 | No | Bilaterally absent | 320.5 | Highly malignant | no | yes |
| 12 | 6 | No | Not checked | Not checked | Not checked | no | Not checked |
